# Past, present and future of psychiatry

**DOI:** 10.1192/j.eurpsy.2021.69

**Published:** 2021-08-13

**Authors:** N. Sartorius

**Affiliations:** Mental Health, Association for the Improvement of Mental Health Programs, Geneva, Switzerland

**Keywords:** The future of psychiatry, primary prevention of mental disorders

## Abstract

Over the past few decades, psychiatry and mental health sciences have reached several major goals. The importance of mental health and the huge contribution to the burden of disability produced by mental and neurological disorders has been recognized by all and most recently also by the United Nations. Treatment technology has developed and permits the effective management of most mental disorders. Progress has also been made in the recognition of human rights of people with mental illness and those who care for them. More has to be done in these areas but there are also new tasks that are before psychiatry. These include the addition of primary prevention of mental disorders to previous efforts to ensure secondary and tertiary prevention of mental health problems; the development of appropriate ways of work in order to cope with problems of comorbidity of mental and physical disorders; and a fundamental reorientation of training in psychiatry and related sciences.

**Disclosure:**

No significant relationships.

